# Pregnancy Outcome of Women with Chronic Hepatitis B who Discontinued Antiviral Treatment before or in the Early Pregnancy

**DOI:** 10.7150/ijms.38410

**Published:** 2020-01-01

**Authors:** Xuesong Gao, Xuefei Duan, Haodong Cai, Yuhong Hu, Min Liu, Kai Kang, Mingfang Zhou, Dong Fu, Wei Yi

**Affiliations:** 1 Department of General Medicine, Beijing Ditan Hospital, Capital Medical University, Beijing, China.; 2 Hepatology clinic, Beijing Ditan Hospital, Capital Medical University, Beijing, China.; 3 Department of Obstetrics and Gynecology, Beijing Ditan Hospital, Capital Medical University, Beijing, China.

**Keywords:** chronic hepatitis B, pregnancy complications, infant, antiviral agents, alanine transaminase

## Abstract

**Background:** The aim of this study was to describe biochemical, virological features and Mother-to child-transmission (MTCT) rate in chronic hepatitis B (CHB) women who stopped antiviral therapy before or in the early pregnancy.

**Methods:** This was a single-center, retrospective study. Forty-three CHB women who stopped treatment before or in the early pregnancy and 103 CHB women with tenofovir disoproxil fumarate (TDF) treatment throughout pregnancy were enrolled. The virological and biochemical flares during pregnancy and postpartum period were studied. MTCT rates were also compared.

**Results:** During pregnancy, ALT flares (43.9% vs 1.0%) and viral rebound (31.7% vs 0) were more common in women who stopped treatment (P<0.001). Postpartum ALT flares were less frequent in women with treatment than those stopped treatment (0 vs 6/35, P = 0.001). The birth defect rate in the mothers who stopped treatment did not statistically differ from that of mothers treated throughout pregnancy (4.9 % vs 3.9 %, P = 1.000). There were no significant differences of gestational complications between the two groups, except intrahepatic cholestasis of pregnancy (12.2% vs 0, P = 0.002). The rate of MTCT in mothers who discontinued treatment was higher (2.4% vs 0, P = 0.285), although there was no statistically significant.

**Conclusion:** ALT flares were common in mothers who discontinued antiviral therapy. Thus, these pregnant women should be monitored closely. Cessation of treatment was not recommended although no hepatic failure was observed. Larger studies are needed to evaluate the safety of discontinuation before pregnancy.

## Introduction

There were approximately 257 million persons with chronic hepatitis B in the world, including 65 million women of childbearing age who can potentially transmit HBV to their babies 1. Mother-to child-transmission (MTCT) remains an important mode of viral transmission in high prevalence areas 2. An association between high maternal levels of HBV viral load and increased risk of MTCT has been identified. The China national hepatitis B sero-epidemiologic surveys in 2006 showed that the prevalence of hepatitis B surface antigen (HBsAg) positivity among childbearing women was 6.6% 3. Typically, antiviral treatment is not indicated in women of childbearing age because they are more likely to be in the immune-tolerant phase. Therefore, the current guideline recommends starting antiviral therapy for women in the immune-tolerant phase with HBV DNA > 2 × 10^6^ IU/mL during the third trimester of pregnancy to further reduce the risks of perinatal transmission 4. However, patients with significant liver injury who meet the standard indications for HBV therapy should consider the antiviral treatment. If a woman becomes pregnant during long-term oral nucleos(t)ide analogues (NAs) treatment, careful consideration needs to be made on whether to stop or to continue antiviral therapy. Previous studies demonstrated that antiviral therapy throughout pregnancy was safe 5-8. However, some women choose to discontinue therapy concerning the safety of medications. There is no consensus on safe cessation of long-term NAs therapy. With relatively longer consolidation therapy, safe discontinuation of NAs seems to be a feasible way in a substantial proportion of patients 9, 10. Nevertheless, treatment cessation could also lead to serious complications and even death in a few patients with advanced fibrosis or cirrhosis 11. Kim et al investigated 12 pregnant women on antiviral therapy discontinued drugs as soon as the awareness of the pregnancy to avoid utero exposure. They reported 66.7% patients (8/12) had a virological relapse, 50% (6/12) had severe ALT flares. All patients recovered without an event of hepatic decompensation 12. However, limited information is available in pregnant women with chronic hepatitis B (CHB).

Therefore, we evaluated the pregnancy outcomes of withdrawal of antiviral treatment before pregnancy in women with CHB.

## Methods

### Study population

We performed a retrospective study of consecutive CHB women evaluated during pregnancy in Beijing Ditan Hospital, Captital Medical University between 2010 and 2018. Patients were diagnosed with CHB according to AASLD guideline.^4^ Women were excluded if they met any of the following criteria: evidence of hepatocellular carcinoma, or hepatic decompensation; systemic diseases such as cardiopulmonary, neurological or renal disease; co-infection with human immunodeficiency virus, hepatitis C virus, hepatitis D virus, toxoplasmosis, syphilis, rubella or cytomegalovirus; history of amniocentesis during pregnancy; a familial history of genetic disease in either member of a couple; previous delivery of a child with a deformity.

They were divided into two groups. The pregnant women who stopped antiviral treatment by themselves were included in Group 1. They discontinued the antiviral therapy after a careful consideration of the risks of hepatitis flares and HBV MTCT against fetal exposure during the pregnancy. Group 2 included patients who had tenofovir disoproxil fumarate (TDF) treatment throughout pregnancy. Ethical approval for this study was granted by the institutional review board of Beijing Ditan Hospital, which waived the need for informed consent for this observational study.

We abstracted data from obstetric records of Beijing Ditan Hospital. Information included maternal demographic data, serum alanine transaminase (ALT) levels, serum creatinine (Cr), HBV serologic markers, HBV DNA levels, pregnancy complications, gestation time (weeks), delivery mode. The following infant data were collected: birth weight, birth length, Apgar score (1 minute) at birth, infant adverse events, birth defect, HBV virological testing at birth and after completion of the three hepatitis B vaccination doses.

### Immunoprophylaxis schedule and laboratory tests

All infants were given standard immunoprophylaxis: 200 IU HBIG (Chengdu Rongsheng Pharmaceuticals, Chengdu, China) and 10 μg recombinant hepatitis B vaccine (Hansenula Polymorpha, Dalian Hissen Bio-pharm, Dalian, China) injected within 2 hours after birth. The vaccine was boosted at 1 and 6 months after birth. The serum of infants was collected before vaccination and HBIG injection at birth and at 7 months after birth.

All of the parameter testing was performed in the laboratory of Beijing Ditan hospital. HBsAg, hepatitis B surface antibody (anti-HBs) and HBeAg were measured using a chemiluminescent microparticle immunoassay (Architect i2000 analyser; Abbott Diagnostics, Abbott Park, IL, USA). The serum HBV DNA level was quantified using real-time polymerase chain reaction (PCR) (Shanghai Kehua Bioengineering Co. Ltd., Shanghai, China), with a lower limit of 100 IU/mL. The ALT level was tested using a Hitachi 7600 fully automatic biochemical analyser (Wako Pure Chemical Industries, Ltd., Tokyo, Japan, ULN ≤ 40U/L). Serum creatinine (normal 0.46-0.83 mg/dl), CK (normal 40-200 U/L), and phosphorus (normal 2.74-4.88 mg/dl) were measured using an automatic analyser (Hitachi 7600- 020, Hitachi High Technologies Co., Tokyo, Japan).

### Outcomes assessment

The primary outcomes were: miscarriage, premature (<37w), and birth defect. Other outcomes included preeclampsia, gestational diabetes (GDM), placenta previa, placental abruption, and preterm premature rupture of the membranes; neonatal outcomes for singleton pregnancy included weight, length and 1- min Apgar score; the rates of HBV MTCT, defined by HBsAg seropositivity or HBV DNA positivity at 7 months.

### Statistical methods

Continuous variables normally distributed were expressed as mean ± standard deviation (SD) and compared by Student's t test. Quantitative data non-normally distributed were presented as median and range and compared by Man-Whitney U test. Categorical variables were presented as numbers and percentages and compared by chi-square analysis or Fisher' s exact test. P < 0.05 was considered statistically significant. All data were analyzed using SPSS (version 22.0; IBM Corp Ltd., Armonk, NY).

## Results

### Baseline characteristics

During the period from January 1, 2010 through June 30, 2018, a total of 147 women with active CHB were retrospectively enrolled at 12 weeks of gestational age. Maternal characteristics and outcomes are summarized in Table 1. In Group 1, there were 43 women with 43 pregnancies and 41 live births. They discontinued antiviral therapy before or during first trimester. A total of 110 pregnancies in 104 CHB women were included in Group 2. There were 103 live births. All women became pregnant while receiving TDF treatment and continued their TDF treatment throughout pregnancy. In Group 1, six women discontinued antiviral treatment at the median gestational age of 5 weeks and 37 stopped treatment before pregnancy. The pregnant women were older in Group 1 than those in Group 2 (29.5 ± 3.4 years vs 30.9 ± 3.8 years, P = 0.042). In Group 1, one pregnant woman had stillbirth and one was induced because of dead fetus. There were 3 cases of stillbirths, 3 cases of miscarriages and one case of induced labour in Group 2. The rate of live birth in mothers who discontinued antiviral therapy was similar with that in mothers on TDF treatment (41/43 or 95.3% vs 103/110 or 93.6%, P = 0.982).

At baseline, HBV DNA was higher in women who had already discontinued therapy as compared with those on treatment (5.1 ± 1.9 log10 IU/mL vs 3.6 ± 0.9 log10 IU/mL, P = 0.010). Most women were HBeAg-positive (35/43, 81.4% vs 85/104, 81.7%, P = 0.962). ALT was also higher in women who stopped treatment (35.9 U/L vs 20.4 U/L, P < 0.001), whereas serum creatinine (Cr) was higher in women on TDF treatment (0.51 mg/dl vs 0.55 mg/dl, P = 0.001).

### Maternal and infant safety

The mean gestational weeks of the mothers who discontinued treatment were shorter than the mothers with TDF treatment throughout pregnancy (38 vs 39 weeks, P = 0.005). There were no significant differences in the rate of infants' weights, heights, Apgar scores (1 min) or cesarean rates between the two groups. In Group 1, one infant was diagnosed with renal duplication and one infant was diagnosed with congenital aortic dysplasia. Four cases of birth defects were found at birth in Group 2: one case of right-side cryptorchidism, one case of congenital localized absence of skin, one case of G6PD deficiency. Another case of birth defect was diagnosed with mitochondrial respiratory chain complex I deficiency at the 7-month follow-up. The birth defect rate in Group 1 did not statistically differ from Group 2 (2/41 or 4.9 % vs 4/103 or 3.9 %, P = 1.000) (Table 2). There were no significant differences of adverse birth outcomes between the two groups, except the rate of intrahepatic cholestasis of pregnancy (5/41,12.2% vs 0, P = 0.002) (Table 3). Elevated Cr levels were more frequent in TDF-treated versus mothers with off-treatment (0.58 ± 0.09 mg/dl vs 0.54 ± 0.08 mg/dl, P = 0.047), though the difference was not assessed as clinically significant.

### ALT flares and HBV DNA levels during pregnancy

A viral rebound of increase in HBV DNA > 2 log10 IU/mL was found in 13 women (31.7%). Among women who discontinued treatment, ALT flares (120.5 U/L to 694.6 U/L) were observed in 43.9% (18/41) of women and an increase in HBV DNA of > 2 log IU/mL was observed in 38.9% (7/18) of women. It should be noted that three woman who discontinued treatment before pregnancy had ALT flares during the second trimester and achieved undetectable serum HBV DNA (< 100 IU/mL) before delivery. None of women developed hepatic failure or hepatic decompensation. Resolution of ALT flares occurred spontaneously in 38.9% (7/18) of women. Improved ALT was observed in 22.2% (4/18) of women, in whom ALT decreased to nearly normal values (<2 × ULN).

ALT flares were less likely to occur in women on TDF treatment throughout pregnancy than those in women who stopped treatment (1/103 or 1.0% vs 18/41 or 43.9%, P < 0.001). The other women remained normal to mildly elevated ALT (8 to 87 U/L). No HBV DNA rebound occurred. Most of women (99/103) achieved HBV DNA undetectable (< 100 IU/mL) and the other four women maintained low HBV DNA levels (< 2000 IU/mL). No viral rebound observed (0 vs 31.7%, P < 0.001).

### Efficacy assessment for mothers and infants

At delivery, the median HBV DNA level for mothers in Group 1 was significantly higher than that in Group 2 (6.0 ± 1.4 log10 IU/mL vs 2.3 ± 0.4 log10 IU/mL, P < 0.001). Although the levels of ALT were higher and Cr were lower in the mothers in Group 1 when compared with the mothers in Group 2, all the mothers had normal ALT, CK, Cr and phosphorus at delivery (Table 4). Virological features and MTCT rates of infants in two groups are presented in Table 5. At birth, the infants in the two groups had similar rates of peripheral blood HBsAg positivity (9/41 or 22.0% vs 17/103 or 16.5%, P = 0.443). The levels of HBV DNA were undetectable in the two groups. At 28 weeks of age, one infant born to mother who stopped treatment was positive for serum HBsAg and HBV DNA (1/41 or 2.4%). The MTCT rate of HBV in the mothers who discontinued treatment was higher than the mothers with TDF treatment throughout pregnancy (1/41 or 2.4% vs 0, P = 0.285), although there was no statistically significant.

### ALT flares and HBV DNA levels at postpartum 28 weeks

Six women who discontinued treatment in Group 1 and 21 mothers who treated throughout pregnancy in Group 2 were excluded from the analysis of postpartum flares for insufficient data. No ALT flares were observed at postpartum 28 weeks in women with TDF treatment throughout pregnancy. The frequency of ALT flares was significantly lower in women with TDF treatment throughout pregnancy than in mothers who discontinued treatment (0 vs 6/35 or 17.1%, P = 0.001). One new ALT flares were observed during postpartum in women untreated throughout pregnancy (n=35). Fourteen mothers reinitiated antiviral therapy during postpartum 1-3 months, of whom 11 mothers had viral suppression at postpartum 28 weeks and none achieved HBeAg seroconversion. Among those women who did not reinitiate treatment, one who was HBV DNA undetectable before delivery achieved HBeAg seroconversion before 28 weeks postpartum. Furthermore, one achieved HBeAg seroconversation spontaneously 2 years postpartum later.

The mothers with TDF treatment throughout pregnancy had lower levels of viremia (2.7 ± 0.3 log IU/mL vs 5.5 ± 1.5 log IU/mL, P < 0.001) and ALT levels (18.8 U/L vs 38.2 U/L, P < 0.001) as compared with those mothers who discontinued antiviral therapy at 28 weeks postpartum (Table 6). Among mothers who received antiviral treatment throughout pregnancy, one achieved HBeAg seroconversation and stopped treatment one month postpartum. She remained HBV DNA undetectable and HBeAg negtive at 28 weeks postpartum. One mother achieved HBsAg seroconversation one and half years postpartum.

## Discussion

Cessation of NAs is generally not recommended among CHB patients due to the risk of viral relapse and clinical exacerbation. Similarly, the safety of ceasing therapy needs to be carefully considered for the increased risk of viral rebound and hepatitis flares among pregnant women. However, the recent studies revealed although virological relapses occurred in the majority of off-treatment patients, they are not necessarily followed by ALT elevations and the probability of liver decompensation was low 10, 13. Therefore, discontinuation of treatment before pregnancy maybe a feasible option, especially to a woman with younger age, undetectable HBV DNA levels, low serum HBsAg levels, without advanced fibrosis, and the long duration of on-therapy virological remission. However, little data is available regarding the potential adverse pregnancy outcomes.

The previous studies had focused on the maternal safety. ALT flares during pregnancy and postpartum were observed and they did not evaluate rates of MTCT or the safety of infants 12, 14. In current study, the birth defect rate among mothers who discontinued treatment was not significantly higher when compared with mothers with TDF treatment throughout pregnancy (4.9% vs 3.9%, P = 1.000). The congenital abnormality rates for infants born to mothers with HBV infection were reported to be 1.1%-10.9% 15, 16. Our finding was consistent with these previous studies. Furthermore, renal duplication and congenital aortic dysplasia are congenital malformation. Its aetiology remains unknown and it does not seem to be associated with HBV in pregnancy. Hepatitis flares during the pregnancy or postpartum periods were reported to be linked to maternal mortality 17. The increasing corticosteroid levels to prevent rejection could explain much of the viral relapses and ALT flares 5. The other interpretation issue is the discontinuation of NAs treatment. Fortunately, the clinical outcomes were not severe and none of pregnant women developed hepatic failure in our study, although virological rebound and hepatic flares were common.

No significant differences were noted in the rates of most adverse pregnancy outcomes. An exception is the prevalence of intrahepatic cholestasis (ICP) of pregnancy. A systematic review and meta-analysis from UK to quantify the adverse pregnancy outcome in women with ICP found that serum bile acids of 100 µmol/L or more was association with increased risk of stillbirth in women with ICP and singleton pregnancies (Hazard ratio 30.50 [95% CI 8.83-105.30]) 18. Hu et al showed the rates of HBV infection in the new-borns, fetal distress, neonatal asphyxia, and birth defects, and infant Apgar scores were higher in ICP pregnancies with HBV than the control groups (P < 0.05) 19. No association was reported between ICP and antiviral therapy to date. We inferred that antiviral treatment improved liver function, therefore, reduced the risk of ICP.

This study has several limitations. It was retrospective study and not all patients had sufficient data. In fact, we do not recommend to stop antiviral treatment before pregnancy regarding to the infant safety. The decisions to stop treatment should be considered carefully and individualized. In addition, the clinical implications have been limited by the small sample size.

In conclusion, nearly half of women who stopped antiviral treatment experienced ALT flares and viral rebounds throughout the course of their pregnancy. However, all patients recovered during pregnancy. We do not recommend to stop antiviral treatment before pregnancy. The women who withdraw antiviral treatment before pregnancy should be monitored closely during pregnancy and postpartum.

## Figures and Tables

**Table 1:** Baseline demographic and clinical characteristics of pregnant women

Variable	Group 1	Group 2	P
Mothers (n)	43	104	
Pregnancies (n)	43	110	
Live births, n (%)	41 (95.3)	103 (93.6)	0.982
Age (years)	29.5 ± 3.4	30.9 ± 3.8	0.042
HBeAg positive, n (%)	35 (81.4)	85 (81.7)	0.962
ALT (U/L)	35.9 (25.0-77.6)	20.4 (15.2-26.7)	< 0.001
CK (U/L)	43.3 (34.1-81.2)	61.1 (46.3-78.7)	0.094
Cr (mg/dl)	0.51 (0.47-0.56)	0.55 (0.51-0.62)	0.001
Phosphorus (mg/dl)	3.5 (3.1-3.8)	3.4 (3.1-3.7)	0.729
HBV DNA (log_10_ IU/mL)	5.1 ± 1.9	3.6 ± 0.9	0.010

Values presented as median (range) or mean ± SD. ALT, alanine aminotransferase; CK, creatine kinase; Cr, serum creatinine; HBeAg, hepatitis B e antigen; HBV, hepatitis B virus.

**Table 2:** Neonatal clinical characteristics

Variable	Group 1 (n=41)	Group 2 (n=103)	P
Apgar (1 min)	10.0 (10.0-10.0)	10.0 (10.0-10.0)	0.116
Weight (kg)	3.4 (3.2-3.6)	3.3 (3.1-3.5)	0.051
Length (cm)	50.0 (50.0-50.0)	50.0 (50.0-50.0)	0.054
Gestational age (weeks)	38.0 (37.5-39.0)	39.0 (38.0-40.0)	0.005
Caesarean section, n (%)	22 (53.7)	49 (47.6)	0.510
Birth defect, n (%)	2 (4.9)	4 (3.9)	1.000

**Table 3:** Complications and adverse events of pregnancy

Variable	Group 1 (n=41)	Group 2 (n=103)	P
Premature birth, n (%)	3 (7.3)	3 (2.9)	0.464
Postpartum haemorrhage, n (%)	1 (2.4)	4 (3.9)	1.000
Oligohydramnios, n (%)	2 (4.9)	8 (7.8)	0.801
Gestational diabetes mellitus, n (%)	14 (34.1)	20 (19.4)	0.060
Meconium staining of the amniotic fluid (III degree), n (%)	2 (4.9)	6 (5.8)	1.000
Premature rupture of membranes, n (%)	7 (17.1)	11 (10.7)	0.295
Gestational hypertension, n (%)	2 (4.9)	0 (0.0)	0.080
Pre-eclampsia, n (%)	1 (2.4)	0 (0.0)	0.285
Hypothyroidism, n (%)	3 (7.3)	4 (3.9)	0.663
Intrahepatic cholestasis of pregnancy, n (%)	5 (12.2)	0 (0.0)	0.002
Placenta previa, n (%)	1 (2.4)	3 (2.9)	1.000

**Table 4:** Changes in HBV DNA and biochemical markers at delivery for the two groups

Variable	Group 1 (n=41)	Group 2 (n=103)	P
ALT (U/L)	25.6 (16.9-56.7)	16.1 (12.5-20.5)	< 0.001
CK (U/L)	60.3 (35. 3-87.7)	57.4 (48.9-81.0)	0.764
Cr (mg/dl)	0.54 ± 0.08	0.58 ± 0.09	0.047
Phosphorus (mg/dl)	3.4 ± 0.5	3.5 ± 0.5	0.363
HBV DNA (log_10_ IU/mL)	6.0 ± 1.4	2.3 ± 0.4	< 0.001

ALT, alanine aminotransferase; CK, creatine kinase; Cr, serum creatinine; HBV, hepatitis B virus.

**Table 5:** Virological features and MTCT rates of infants in TDF treated group and untreated group.

Variable	Group 1 (n=41)	Group 2 (n=103)	P
**At birth**			
HBsAg+, n (%)	9 (22.0)	17 (16.5)	0.443
HBV DNA ≥100 IU/mL, n (%)	2 (4.9)	0 (0.0)	0.079
**Postpartum 28 weeks**			
HBsAg+, n (%)	1 (2.4)	0 (0.0)	0.285
HBV DNA ≥100 IU/mL, n (%)	1 (2.4)	0 (0.0)	0.285

**Table 6:** HBV DNA and biochemical markers at 28 weeks postpartum for the two groups

Variable	Group 1 (n=35)	Group 2 (n=82)	P
ALT	38.2 (20.6-100.8)	18.8 (14.7-21.7)	< 0.001
HBeAg, n (%)	25 (71.4)	51 (62.2)	0.338
HBV positive, n (%)	20 (57.1)	3 (3.7)	< 0.001
HBV DNA (log_10_ IU/mL)	5.5 ± 1.5	2.7 ± 0.3	< 0.001

ALT, alanine aminotransferase; CK, creatine kinase; Cr, serum creatinine; HBV, hepatitis B virus.
